# Metformin targets a YAP1-TEAD4 complex via AMPKα to regulate CCNE1/2 in bladder cancer cells

**DOI:** 10.1186/s13046-019-1346-1

**Published:** 2019-08-27

**Authors:** Yanju Wu, Qianqian Zheng, Yan Li, Guang Wang, Shuting Gao, Xiaodong Zhang, Xu Yan, Xinwen Zhang, Jisheng Xie, Yuanyuan Wang, Xun Sun, Xin Meng, Bo Yin, Biao Wang

**Affiliations:** 1 Department of Biochemistry and Molecular Biology, School of Life Sciences, China Medical University, No.77 Puhe Road, Shenyang North New Area, Shenyang, Shenyang, 110122 Liaoning Province China; 2 Department of Pathophysiology, College of Basic Medical Science, China Medical University, No.77 Puhe Road, Shenyang North New Area, Shenyang, Shenyang, 110122 Liaoning Province China; 3 Department of General Surgery, the Fourth Affiliated Hospital of China Medical University, 4 Chongshan East Street, Huanggu District, Shenyang, Shenyang, 110032 Liaoning Province China; 4 Department of Laboratory Animal Science, China Medical University, No.77 Puhe Road, Shenyang North New Area, Shenyang, 110122 Liaoning Province China; 5 Department, School & Hospital of Stomatology, China Medical University, No.117 North Avenue Nanjing, Heping District, 110002, Shenyang, 110002 Liaoning Province China; 6 Center of Implant Dentistry, School & Hospital of Stomatology, China Medical University, No.117 NanJing North Street, HePing District Shenyang, Shenyang, 110002 Liaoning Province China; 7 Department of Histology and Embryology, Youjiang Medical College for Nationalities, No. 98, Chengxiang Road, Youjiang District, Baise City, Guangxi Province China; 8 Department of anesthesiology, the Fourth Affiliated Hospital, China Medical University, No. 4 Chongshan East Road, Yuhong District, Shenyang, 110032 Liaoning Province China; 9 Department of Immunology, College of Basic Medical Sciences of China Medical University, No.77 Puhe Road, Shenyang North New Area, Shenyang, 110122 Liaoning Province China; 10 Department of Urology, ShengJing Hospital of China Medical University, No.36 SanHao Street, HePing District Shenyang, Shenyang, 110004 Liaoning Province China

**Keywords:** Yes-associated protein 1, Cyclin E, TEAD4, Bladder cancer, Metformin

## Abstract

**Background:**

Metformin has been reported to function as the anti-tumor inhibiting the growth of different types of cancers, including bladder cancer. But there are few reports on the roles of Yap1, the key molecule of Hippo pathway, in the metformin induced inhibition of bladder cancer (BLCA). We are wondering if the inhibitory effect of metformin on bladder cancer is fulfilled via Yap1 and exploring the related mechanism.

**Methods:**

MTS and colony formation assays were used to explore the cellular viabilities and proliferation of BLCA cells challenged by metformin at different concentrations, in vitro. Flow Cytometry (FCM) was used to analyze the cell cycle and the cellular apoptosis of the BLCA cells. Western Blot was performed to detect the expressions of AMPKα, Yap1, CCND1, CCNE1/2 and CDK2/4/6 in the metformin-treated BLCA cell lines. RNAi method was used for the related genetic functional analysis. The relationships among Yap1, TEADs and CCNE1/2 were predicted and evaluated using bioinformatics, dual-luciferase reporter and co-immunoprecipitation (Co-IP) assays. For in vivo experiments, a xenograft model was used to investigate the effects of metformin on the proliferation of BLCA cells. And Immunohistochemistry (IHC) assay was performed to assess the expressions of CCNE1/2 and Yap1 proteins in the tumor tissues from the model.

**Results:**

Metformin could inhibit the proliferation of the BLCA cells via inducing the G1 cell cycle arrest without apoptosis. And metformin upregulated the phosphorylated AMPKα and decreased the expressions of Yap1 and CCND1, CCNE1/2 and CDK4/6. AMPK inhibition by compound C (CC) restored the cell proliferation and the G1 cell cycle arrest induced by metformin, in vivo. Knockdown of YAP1 inhibited the proliferation of BLCA cells and caused the cell cycle arrest at G1 phase by decreasing the expressions of CCNE1/2 and other G1 phase related molecules, which has been restored by the Yap 5SA mutant. Bioinformatics analysis showed that trans-factor TEAD4 was highly expressed and positively associated with the expressions of CCNE1 and CCNE2 in BLCA and only TEAD4 was precipitated by Yap1 in the BLCA cells. Further studies demonstrated that Yap1 positively regulated both CCNE1 and CCNE2 expressions via forming complex with TEAD4. Furthermore, we observed that metformin inhibited the cell proliferation by decreasing the expressions of Yap1 and both CCNE1 and CCNE2 in xenograft model.

**Conclusions:**

The results of our study reveal a new potential regulatory pathway in which metformin inhibits cell proliferation via AMPKα/Yap1/TEAD4/CCNE1/2 axis in BLCA cells, providing new insights into novel molecular therapeutic targets for BLCA.

**Electronic supplementary material:**

The online version of this article (10.1186/s13046-019-1346-1) contains supplementary material, which is available to authorized users.

## Background

Bladder cancer (BLCA) is the most frequent cancer of the urinary tract, ranking the 7th most common cancer in men and the 17th in women. Upon initial diagnosis, approximately 75–80% of cases are diagnosed as non-muscle invasive bladder cancer (NMIBC) and the remaining as muscle-invasive bladder cancer (MIBC). The standard treatment strategy for patients with MIBC is the radical cystectomy, but about half of patients diagnosed with MIBC have imperceptible metastases at the time of treatment of the primary tumor. Although peri-operative chemotherapy was introduced, the five-year recurrence and progression rates can add up to 31–78% and 1–45% respectively [1] after the first treatment. Therefore, finding a therapeutic strategy of preventing bladder carcinoma progression and recurrence is required urgently.

It’s well known that the deregulated cellular energetic metabolism is a key hallmark of cancer, and the metabolic reprogramming in cancer has been the focus of cancer research over the last decade. As an important enzyme in maintaining cellular energy homeostasis, AMP dependent protein kinase (AMPK) is a highly-conserved serine/threonine protein kinase consisting of catalytic (α) and regulatory (β and γ) subunits and widely expressed in eukaryotes. AMPK plays a central role in regulating metabolic pathways and the fast response of the cell to energetic changes.

AMPK can regulate several signal pathways by inducing phosphorylation of many important cancer-related targets, e.g., mTOR, p53 and Akt et al., ultimately leading to anticancer activities. Although it has been proved that the role of AMPK in cancer is context-dependent [2], in most cases the activation of AMPK inhibits aerobic glycolysis, protein synthesis and proliferation of cancers [3–5]. On the contrary, the knockout of AMPK accelerates the development of lymphomas in MYC oncogene transgenic mice and promotes Warburg effect through stabilization of HIF1α [2]. More and more researches support the notion that AMPK is becoming a possible metabolic tumor suppressor and a potential target for prevention and treatment of many types of cancer, such as lung cancer, colorectal cancer, liver cancer, prostate cancer and melanoma. So, the usage of AMPK agonists for cancer treatment has been proposed in many research work. Many drugs or certain natural compounds can activate AMPK, among which Metformin (MET) is the magic bullet that wildly studied for cancer treatment in different cancer cells.

As one of the most popular drugs used for type 2 diabetes therapy, metformin, an activator of AMPK, has aroused keen interest as a potential anticancer agent and reduces cancer risk and mortality, including bladder cancer [6], which has been proved by many studies [7–11]. It has been reported that metformin can block precancerous lesions progressing to invasive tumors through inhibiting the activation of STAT3 pathway [12]. And metformin showed significant inhibitory effect on bladder tumor growth in syngeneic ortho-topic model through intra-vesical administration. And drug combination with metformin induced a strong anti-proliferative and anti-colony forming effect and apoptosis in bladder cancer cell lines [13]. While, some studies showed that metformin has no considerable inhibitory effect on the recurrence rate of bladder cancer, but that it can delay tumor recurrence [14].

Cyclin E (CCNE), which is required for the transition from G1 to S phase of the cell cycle via its interactions with cyclin dependent kinases (CDK), specifically CDK2, has been implicated in various carcinomas, including pituitary, breast, gastric, ovarian, lung, stomach, colorectal and bladder cancers [15–21]. Some research showed that CCNE levels were inversely correlated with tumor grade and significantly associated with a non-papillary growth pattern and invasiveness of the bladder tumors and poor overall survival [20, 21]. These results suggest that CCNE could be used as a potential therapeutic target and a new prognostic marker for a set of cancers, including the bladder cancer [17, 18, 20–22].

Yes-associated protein (YAP), a transcription co-activator in the Hippo tumor suppressor pathway, plays a central role in regulating cell growth, tissue homeostasis, and organ size. As the key co-effector, YAP1 and its closely related paralog TAZ act as oncogenes in various cancers. It has been reported that the over-expression of YAP in mouse liver reversibly enlarged livers and eventually led to tumor formation [23, 24]. Moreover, the elevated activation of YAP has been observed in various human cancers [25–30]. Yap1 also plays an important role in the development of bladder and the deregulation of Yap1 is significantly associated with the development and metastasis of human bladder cancer [31]. Several studies have shown that Yap1 was dramatically up-regulated in bladder cancer samples at both mRNA and protein level, contributing to progressive features and poor prognosis of bladder cancer [31, 32], and acting as a biomarker for bladder cancer. The emergence of the Yap in BLCA progression may aid in identifying new pharmaceutical targets for BLCA management.

It has been proved that metformin can decrease the total protein expression of Yap and increase the phosphorylation level of Yap [33] in hepatocellular carcinoma cells. And in the glioma cells, cytoplasmic retention was enhanced by metformin, and thus the transcriptional modulating activity of Yap was inhibited [34]. And meanwhile, it has been reported that AMPK can inhibit Yap directly via phosphorylation of Yap S94, disrupting the formation of YAP-TEAD, and indirectly via activation of the Lats kinase [35].

In this study, we found that metformin inhibited the proliferation of BLCA cells by arresting cells at G1 phase through activating AMPK and inhibiting Yap1 expression which resulted in the reduced the expression of CCNE1/2 at transcriptional level directly. The above mechanism studies on AMPK/YAP/CCNE1/2 axis regulation by metformin provide insights into potential approaches with metformin in BLCA therapy.

## Materials and methods

### Cell culture

T24, SW780 and 5637 of bladder cancer cell lines were used in this study. T24 and SW780 were obtained from the American Type Culture Collection (Rockville, MD, USA). T24 and SW780 were grown in McCoy’s 5a Medium Modified and L-15 Medium (Gibco, MD, USA) respectively, supplemented with 10% fetal bovine serum (FBS, Gibco-BRL, MD, USA) at 37 °C in a 5% CO_2_ humidified atmosphere.

### Cell viability assay

To determine the effect of metformin on BLCA cells growth, MTS assay was conducted. Cells were seeded into 96-well plates and various concentrations of metformin was added to a final volume of 100uL of growth medium per well. The cells were incubated with 20uL MTS solution for 4 h at 37 °C. The optical density at 492 nm was read by a multi-scan spectrophotometer.

### Colony formation assay

All the BLCA cells at a density of 1 × 10^3^ were seeded in 6-well plates in culture medium with 10% FBS for 1 week. Then, the cells were fixed with methanol for 30 mins and stained with 1% crystal violet for 10 mins. Colonies of more than 50 cells were counted. All experiments were performed in triplicate. Data were presented as the mean ± SD.

### Flow cytometric analysis

Briefly, the BLCA cells treated with/without metformin were harvested and washed twice with cold phosphate buffered saline (PBS, Gibco Invitrogen), fixed with 75% ethanol and stored at 4 °C. On the day of analysis, the cells were washed and collected using cold PBS in tubes. Propidium iodide (PI) stain containing 10 mg/mL RNase A (100 mg/ml) was added to each tube, which was then incubated at 4 °C for 30 min prior to analysis of cell cycle and cellular apoptosis.

### Immunofluorescence analysis

Cells were fixed for 10 min at room temperature with 75% ethanol in PBS, per-meabilized with 0.3% Triton X-100 in PBS, incubated with the blocking buffer (PBST.

Containing 5% bovine serum albumin), and subsequently probed with anti-YAP, and Alexa Fluor 488-conjugated secondary antibodies (Molecular Probes). Slides were mounted using VectaShield with 4′,6-diamidino-2-phenylindole (DAPI, Vector Laboratories).

### Total RNA isolation and quantitative RT-PCR

Total RNA was isolated with TRIZOL reagent (TaKaRa, Japan) and cDNA were synthesized from 5 μg total RNA using M-MLV Reverse Transcriptase (Promega, WI, USA) with random hexamer priming. Quantitative RT-PCR was performed in the LightCycler 480II system (Roche, Basel, Switzerland) using cDNA Master SYBR Green I dye (Promega, WI, USA) with specific primers. The fold changes were calculated according to the formula 2^-ΔΔCt^ method. Each reaction was performed in triplicate. The corresponding sequences of forward and reverse primers are provided in Additional file 3.

### RNA interference

Cells were transfected with different siRNAs using Lipofectamine 3000 Transfection Reagent (ThermoFisher Scientic). Briefly, cells were seeded in six-well plates and transfected with siRNA and Lipofectamine 3000 Transfection Reagent, which were each incubated separately in Opti-MEM for 5 min, mixed together for 15 min at room temperature, and then the mixture was applied to the cells plated in 1 ml of medium (final siRNA concentration, 80 nM). The siRNA sequences used in this experiment were provided in the Additional file 3.

### Western blotting

Briefly, cells or tissues were lysed in RIPA lysis buffer (Boston Bioproducts) containing protease and phosphatase inhibitors (Roche), and protein concentrations were determined with the BCA protein estimation assay (Thermo Scientific). Equal amounts of protein were separated by SDS-PAGE and transferred to nitrocellulose membrane. Blots were probed with antibodies against total Yap1, TEAD4, CyclinD1, CyclinE1, CyclinE2, CDK2, CDK4, CDK6, p-Yap (127ser), cleaved Caspase 3/7, Bcl-2 and Bax (CST, USA).

### Luciferase assay

T24 and Sw780 cells were plated in 24-well plates and transfected with MMTV-luc containing CCNE1 and CCNE2 promotor sequence using Lipofectamine 3000 (Invitrogen), respectively. The pRL-TK was used as an internal control. Luciferase activity was measured by Dual-Luciferase Assay (Promega, Madison, WI) according to the manufacturer’s manual.

### Chromatin immunoprecipitation (ChIP)

Briefly, protein-DNA complexes were cross-linked by 1% formaldehyde then quenched using 125 mM glycine. Cells were collected in lysis buffer and subjected to sonication. After centrifugation, the supernatant was incubated with Yap1 antibody, and chromatin DNA was purified and subjected to qPCR detection.

### Co-immunoprecipitation protocol

For co-immunoprecipitation, T24 and Sw780 cells were lysed on ice in lysis buffer (20 mM Tris HCl pH 8, 137 mM NaCl, 10% glycerol, 1% Nonidet P-40, 2 mM EDTA and Protease inhibitor) for 30 min with occasional vortexing, followed by a 12-min centrifugation to remove cellular debris. After pre-clearing, 500 μg protein lysate was immunoprecipitated with anti-Yap 1 antibody (CST Rabbit, 1:50 Dilution) and Protein A sepharose beads. Immunoprecipitates were then probed with Anti TEAD4 (abcam- Mouse) and anti Yap1 (CST, Rabbit).

### In vivo studies

Four-to-Five-week-old female nude mice (BALB/c-nu) were obtained from Beijing Vital River Laboratory Animal Technology Co., Ltd. and raised in the Department Laboratory Animal Science at China Medical University. All animal protocols were in accordance with the National Institutes of Health Guide for the Care and Use of Laboratory Animals and was approved by the China Medical University Animal Care and Use Committee. To assess cancer cell proliferation in vivo, T24 cells (1 × 10^6^) were subcutaneously inoculated in axilla of the female nude mice. Six mice were assigned to each group. Tumor height and width were measured with a caliper every 2 days to calculate tumor volume (= width^2^ × height × π/6). For subcutaneous tumor growth, the maximum single tumor cannot exceed 1.5 cm in diameter in mice and no experiments in this study generated tumor burden over this limit. After two weeks of in vivo metformin treatment, primary tumor masses were collected from nude mice, fixed in 4% paraformaldehyde, and embedded in paraffin. For the xenograft studies, the mice were randomly divided into different groups according to ID number.

### Immunohistochemistry

Briefly, the tumor sections were incubated with the PCNA (1:200), Yap1(1:200), CCNE1(1:200), and CCNE2 (1,200) antibodies at 4 °C overnight, respectively; And then incubated with HRP-conjugated antibody for 30 min at 37 °C. After each treatment, the slides were washed three times with TBST for 5 min and were visualized with 3, 3′-diaminobenzidine. Then, sections were dehydrated, cleared, and mounted. Negative controls were prepared by omitting the primary antibody.

### Statistical analysis

All in vitro were performed at least three times. Values are expressed as mean ± standard deviation (SD). The significance of the difference between any two samples was analyzed by t-test using SPSS v13.0; All images were analyzed with ImageJ Software (Version 1.52 k). Values of *p* < 0.05 were considered statistically significant.

## Results

### Metformin inhibits the proliferation of BLCA cells by inducing cell cycle arrest at G0/G1 without apoptosis

**Fig. 1:**
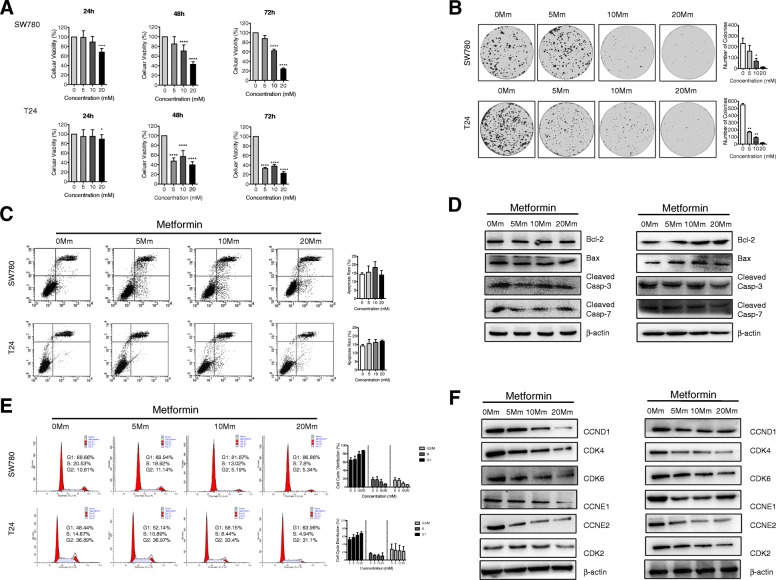
Metformin inhibits cell proliferation via inducing G1 cell cycle arrest in BLCA cells. **a**. Cell viabilities of T24 and Sw780 cells were determined using MTS method when treated with metformin at different concentrations (5 mM, 10 mM and 20 mM) for at 24 h, 48 h and 72 h. **b**. Colony formation assay was carried out to evaluate the proliferation abilities of T24 and Sw780 cells when challenged to metformin at different concentrations. **c**. The cellular apoptosis was analyzed with Flow Cytometry using Annexin V^+^ and PI^+^ staining in the T24 and Sw780 cells treated with/without metformin at 48 h. **d**. Western Blot method was used to detect the expressions of Bcl-2 and Bax, cleaved Caspase 3 and 7. **e**. The cell cycle analysis was performed and compared with Flow Cytometry in T24 and Sw780 cells treated with/without metformin at 48 h. **f**. The key G1 phased related proteins, CCND1, CCNE1/2 CDK4/6, and CDK2 were detected by Western Blot. * means *P* < 0.05, ** means *P* < 0.01, *** stands for *P* < 0.005 and **** stands for *P* < 0.001, compared to the control group

Three human-derived bladder cancer cell lines T24, sw780 and 5637 were used here to determine the effects of metformin on the proliferation of human bladder cancer cells. Each cell line was cultured and treated with metformin at different concentrations (5-20 mM) for 24 h, 48 h and 72 h, respectively. Then the cell viability was determined with MTS assay. At the time point of 24 h, only metformin at 20 mM can inhibit the cell proliferation of T24, SW780 and 5637 (Additional 1: Figure S1A); And after 48 h, ≥10 mM metformin begins to show the inhibitory effects on the cell proliferation in all the BLCA cell lines (Fig. 1a and Additional file 1: Figure S1A), which were comparable with concentrations used in previous studies. To confirm the direct relationship between the decrease in cell viability and the inhibition of cell proliferation, we examined the colony formation capacity of BLCA cells in the presence or absence of metformin for one week. We found that metformin at concentration of 10 mM above can inhibit the colony formation of all the BLCA cells (Fig. 1b and Additional file 1: Figure S1B) efficiently. Both MTS and Colony formation capacity assays showed that metformin decreases the cellular proliferation of BLCA cells.

The effects of metformin on apoptosis and cell cycle were examined with Flow Cytometry to investigate the mechanism(s) by which metformin suppresses tumor growth of T24, SW780 and 5637 cells. First, Annexin V/PI Staining to investigate whether metformin induced the cellular apoptosis in bladder cancer cells. As shown in Fig. 1c and Additional file 1: Figure S1C, the cellular apoptosis is not found in T24, SW780 and 5637 bladder cancer cells treated with different concentrations of metformin, compared with the cells cultured without metformin (0 mM Metformin). In addition, expressions of some vital apoptosis-related proteins were examined with Western Blot. The results showed that metformin could not change the levels of Bcl-2 and Bax in the cell lines (Fig. 1d, Additional file 1: Figure S1D). Caspases play important roles in inducing apoptosis in which caspase-3 and 7 are the executive molecules, so the expressions of activated caspase 3 and 7 were observed with Western Blot. The results showed that there are no significant changes of the cleaved caspase 3 and caspase 7 between the control cells and the cells with metformin treatments (Fig. 1d, Additional file 1: Figure S1D).

And then, the cell cycle of the bladder cancer cells was analyzed and compared after the cells were treated with metformin at different concentrations for 48 h. ≥10 mM metformin treatment obviously induce G1 cell cycle arrest in all the cell lines (Fig. 1e and Additional file 1: Figure S1E). To confirm these data, western blot analysis was used to examine the expression of various key proteins implicated in the transition of the G1 phases in all the cells with and without metformin treatment at the time point of 48 h. The protein levels of cyclin D1 and cyclin E1/2 declined as the concentration of metformin increased and concurrently, CDK4 and CDK6 protein levels decreased progressively in response to metformin in the T24, SW780 cells (Fig. 1f); and similar results were also obtained in 5637 cells (Additional file 1: Figure S1F). And no significant changes to CDK2 have been found in the BLCA cells challenged by MET.

Taken together, these results proved that metformin inhibits the expressions of key proteins related to G1-S transition and induces G1 cell cycle arrest in BLCA cells without inducing apoptosis.

### The activation of AMPKα triggers cell cycle blockage and promotes Yap1 degradation in the BLCA cells challenged against metformin

**Fig. 2:**
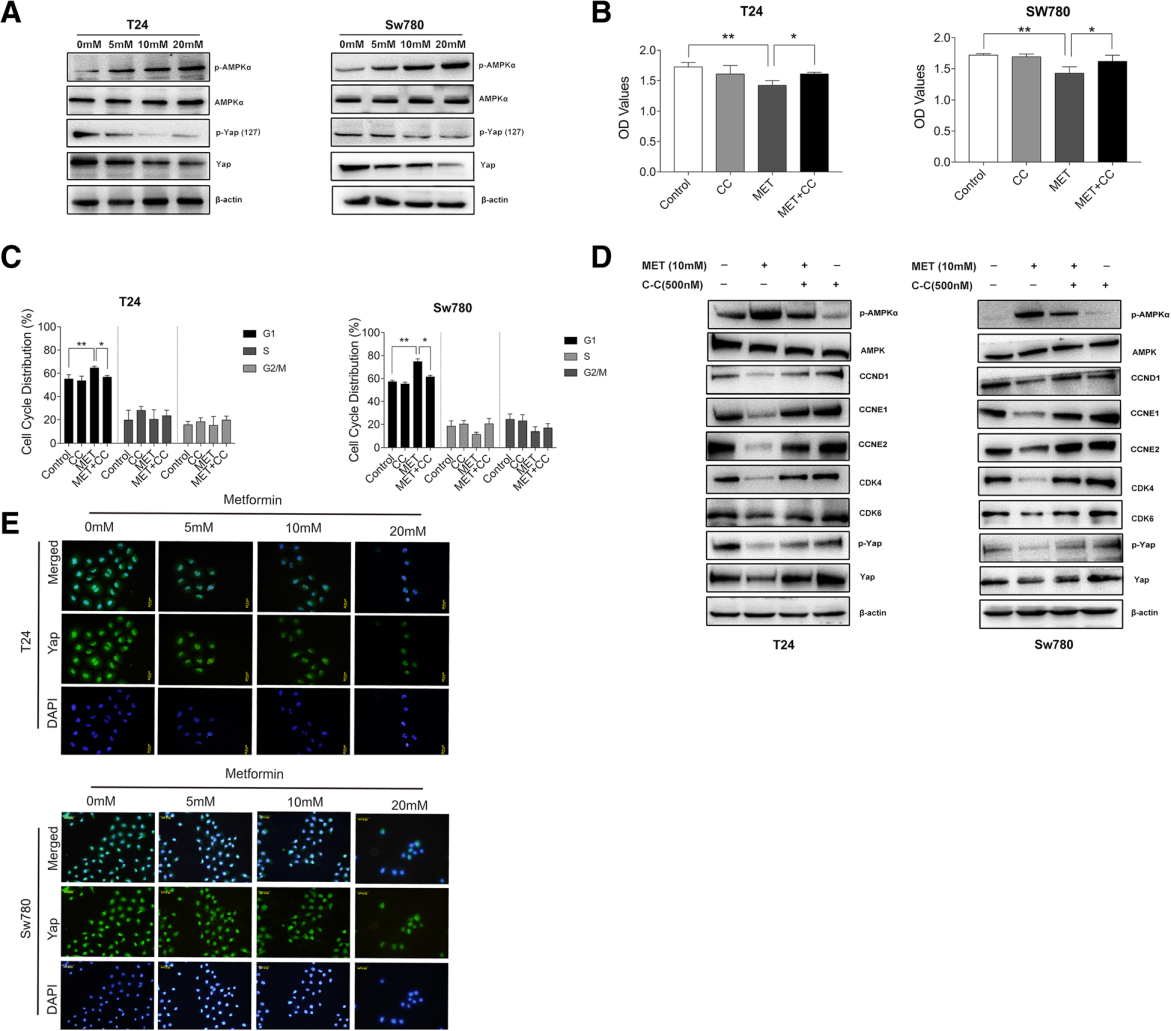
AMPKα plays a central role in cell cycle arrest induced by metformin and promotes Yap1 degradation in the BLCA cells. **a**. Western Blot was used to detect the expressions of AMPK and Yap1 in T24 and Sw780 cells treated with metformin at different concentrations (5 mM, 10 mM and 20 mM). **b**. The cell viabilities of T24 and Sw780 cells were determined when the cells were treated by metformin with/without AMPK inhibitor Compound C (CC) at the time point of 48 h. **c**. The cell cycle analysis was performed using Flow Cytometry when T24 and Sw780 cells were co-treated by metformin with/without CC for 48 h. **d**. The expressions of AMPKα, CCND1, CCNNE1/2 CDK4/6, CDK2 and Yap1 were detected by Western Blot in the T24 and Sw780 cells co-treated by metformin with/without CC for 48 h. **e**. Immunofluorescence was used to examine the overall distribution of Yap1 in the T24 and Sw780 cells challenged by metformin of different concentrations. * means *P* < 0.05 and ** means *P* < 0.01 compared to the control group

Metformin has been proved to inhibit the proliferation of some cancer cells via the activation of the AMPK pathway. Therefore, we investigated if the AMPK pathway was involved in the anti-proliferative effects of metformin on bladder cancer cells. All cells were treated with metformin for 48 h. Western blot analysis revealed that metformin treatment significantly increased AMPK-α phosphorylation on Thr172 in a dose-dependent manner, indicating that metformin induced AMPK activation in the bladder cancer cells (Fig. 2a). But metformin did not change the expression of total AMPK-α at protein level.

To explore the whether the activation of AMPK was involved in the inhibitory effects of metformin on the proliferation of bladder cancer cells, the selective AMPK inhibitor Compound C (CC) was used. The BLCA cells were cultured in the medium containing 10 mM metformin with or without 500 nM CC for 48 h and then MTS method was used to assess the cellular viability. As shown in Fig. 2b, the addition of CC attenuated the inhibitory effects of metformin on the cellular viabilities (*P* < 0.05). And simultaneously, CC could significantly reverse the G1 arrest in cell cycle caused by metformin (Fig. 2c).

Western Blot results (Fig. 2d) showed that CC reversed the increased expression of phosphorylated AMPKα induced by metformin and CC alone decreased the expression of phosphorylated AMPKα without changing the expression of total AMPKα at protein level. Meanwhile, the addition of CC restored the decreased expression of Yap1 induced by MET, which proved that Yap1 expression was negatively correlated with that of phosphorylated AMPKα. Similarly, the decreased expressions of CCND1, CCNE1/2, CDK4 and CDK6 induced by metformin were also restored by the addition of CC.

As the nuclear effector of the Hippo pathway, Yap1 plays an important role in many tumor entities. Here, we evaluated the expression of Yap1 in the BLCA cells treated with metformin and found that metformin decreased the expressions of total Yap1 and Yap1 phosphorylation at Ser127 in a dose-dependent manner (Fig. 2a). We all know that the phosphorylated Yap1 is restrained in the cytoplasm which inhibits its function as transcriptional co-activator. Un-phosphorylated Yap1 can translocate into the nucleus and interact with its nuclear binding partners. To test whether metformin can affect the cellular localization of Yap1 in the bladder cancer cells, we used the Immunofluorescence (IF) method for intracellular translocation processes of Yap1. Yap1 were detected in the cytoplasm and the nucleus of all cells. Yap1 staining was more intensive in the nucleus than in the cytoplasm, and the levels tended to be decreased in the cells treated with metformin (Fig. 2e). Both cytoplasmic Yap1 and nuclear Yap1 were obviously decreased in the cells treated with metformin (Fig. 2e). And the addition of CC also restores the Yap1 expressions in the metformin treated BLCA cells (Fig. 2d), which shows that activation of AMPKα can promote the degradation of Yap1.

From the results above, we concluded that the activation of AMPKα plays a crucial role in the metformin-induced G1 cell cycle arrest, blocking the overall expression of Yap1 without affecting its cytoplasmic/nuclear translocation in the BLCA cells, obviously.

### Yap1 is required in the cell cycle arrest at G1 phase of the bladder cancer cells

**Fig. 3:**
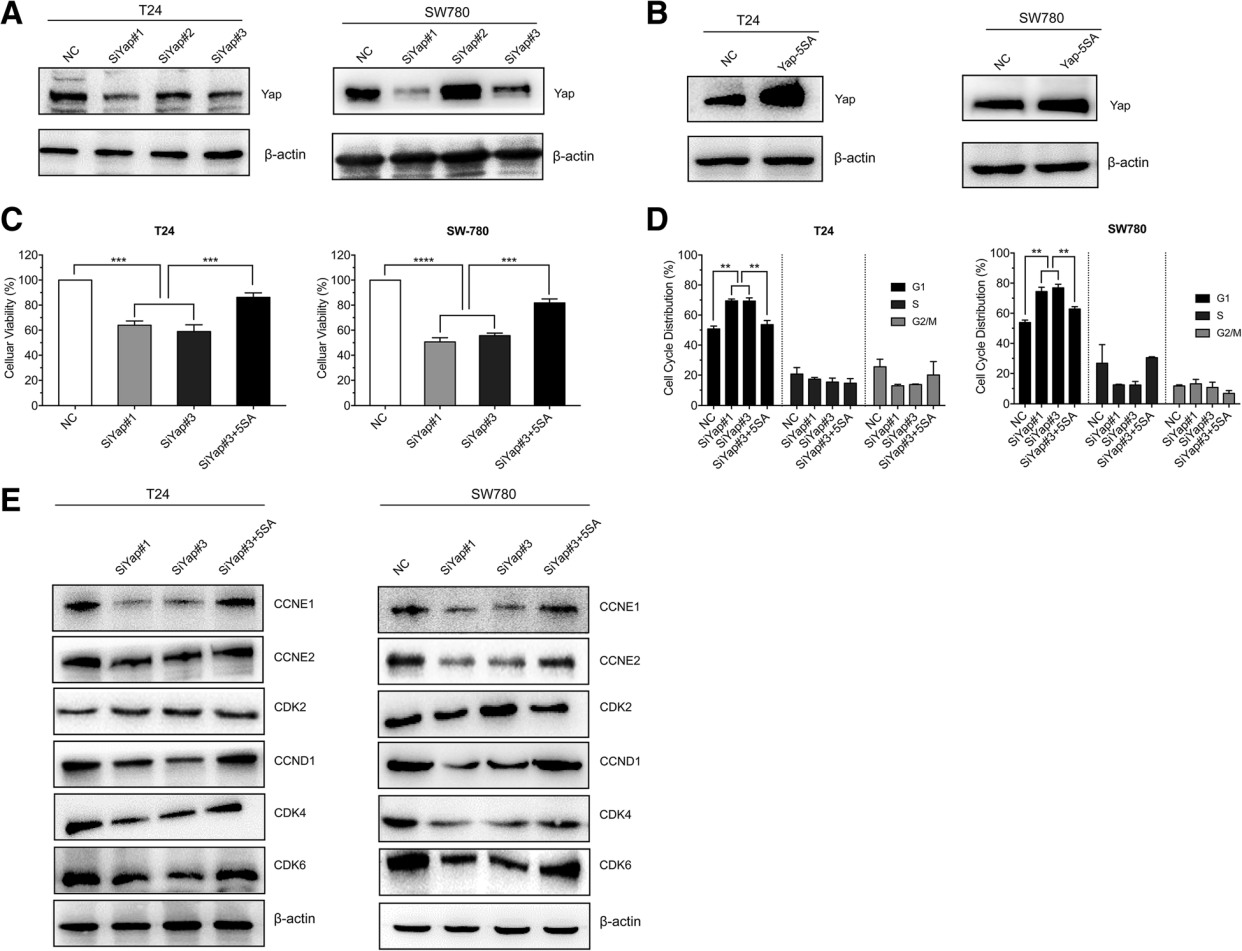
Yap1 knockdown induces the cell cycle arrest at G1 phase in T24 and Sw780 cells. **a**. Yap1-specific small interfering (si) RNA was used to down-regulate the expression of Yap1 and Western Blot method was used to detect the expressions of Yap1 in the T24 and Sw780 cells. **b**. Yap 5SA mutant plasmid (Mutation of all five serine to alanine at S61A, S109A, S127A, S164A, S381A) was transfected into T24 and Sw780 cells to generate a Yap1 mutant resistant to phosphorylation by its up-stream kinases. **c**. the cell viabilities of T24 and Sw780 cells were determined using MTS assay when transfected with Yap1 specific siRNA or Yap 5SA plasmid. **d**. The cell cycle was determined with Flow Cytometry when T24 and Sw780 cells were interfered by Yap1-siRNAs or Yap 5SA plasmid. **e**. The protein expressions of CCNE1/2, CCND1, CDK2 and CDK4/6 were detected in the T24 and Sw780 cells interfered by Yap1-siRNAs or combined transfection with Yap 5SA plasmid. ** means *P* < 0.01 and *** stands for *P* < 0.005, compared to the control group

Yap1 is known to play a role in the development and progression of multiple cancers as a transcriptional regulator and may function as a potential target for cancer treatment. Weather Yap1 was involved in the G1 arrest induced by metformin remains unclear. To evaluate the role of Yap1 in suppression of cellular viability in the bladder cancer cells treated with metformin, first we used RNAi method to knockdown the expression of Yap1 (Fig. 3a) and Yap 5SA mutant plasmid (Fig. 3b) (Mutation of all five serine to alanine at S61A, S109A, S127A, S164A, S381A) to generate a constitutively YAP mutant resistant to phosphorylation by its up-stream kinases. The results from the cell viability assay showed that Yap1 knockdown obviously decreases the cellular viabilities of T24 and SW780 cells and the Yap 5SA expression rescues the cell viabilities loss caused by the Yap1 knockdown (Fig. 3c). And the cell cycle analysis showed that Yap1 knockdown induces G0/G1 cell cycle arrest in T24 and SW780 cells, which can be reversed by the Yap-5SA expression (Fig. 3d). Then, we analyzed the protein levels of cyclin E1/2, cyclin D1 and CDK4/6 with Western Blot. As shown in Fig. 3e, Yap1 knockdown could significantly decreases the CCNE1/2, CCND1 and CDK4/6 expressions at protein level, while expression of YAP-5SA restores their expressions in the T24 and Sw780 cells.

These results strongly suggested that Yap1 could be a central molecule in the metformin induced G1 cell cycle arrest, with inhibition of G1-phase related proteins in the BLCA cells.

### Yap1 has a high possibility to interact with TEAD4 in BLCA cells

**Fig. 4:**
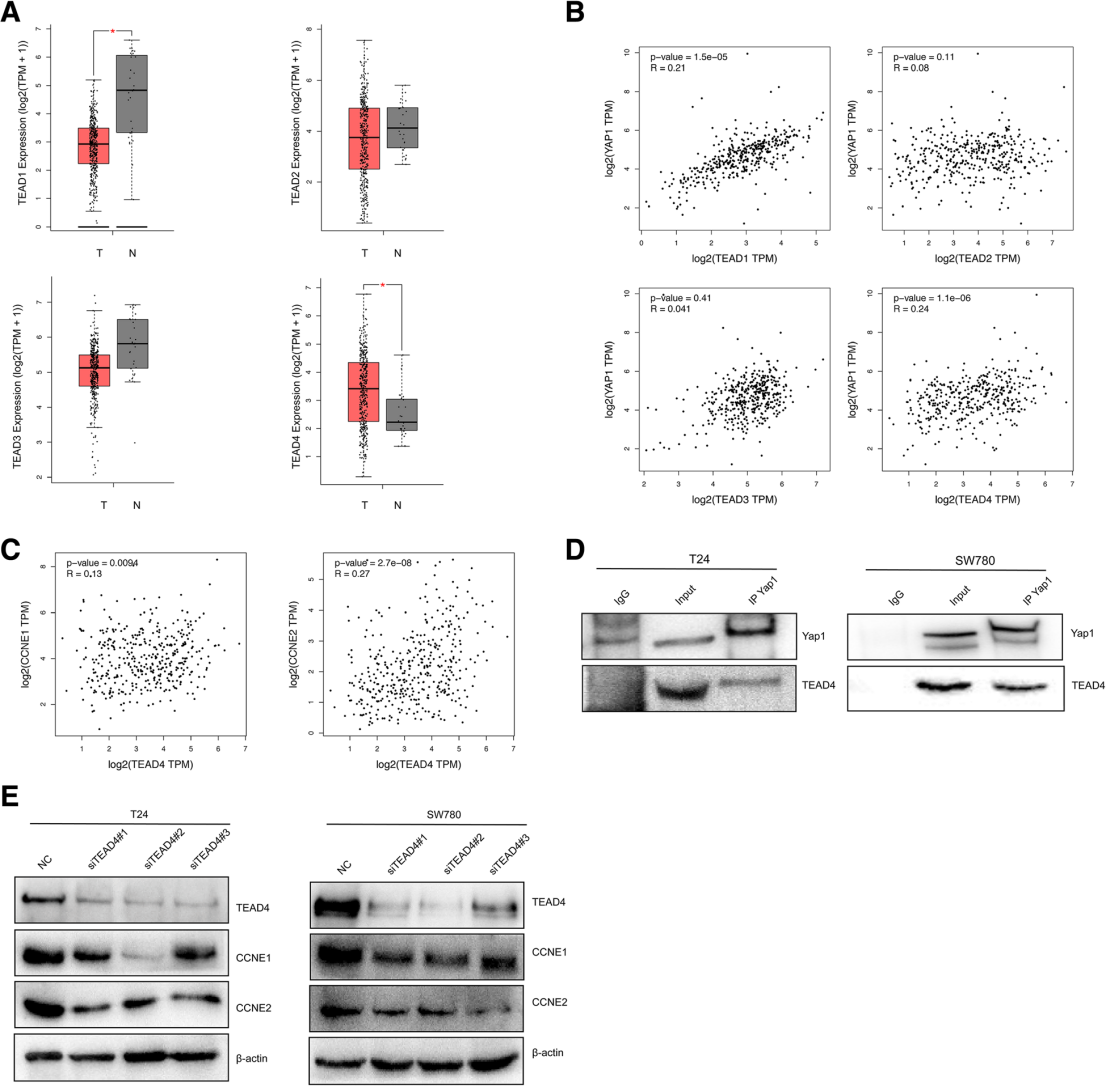
Yap1 interacts with TEAD4 in the BLCA cells. **a**. The expressions of TEAD1–4 in tumor and normal tissues were analyzed and compared using RNA-seq data from TCGA. T stands for tumor tissues (*n* = 404) and N stands for the normal tissues (*n* = 28). **b**. The correlation analysis between TEADs and Yap1 was performed using the RNA-seq data from TCGA. **c**. The analysis of correlation expression between TEAD4 and CCNE1/2 was established using the RNA-seq data from TCGA. **d**. Wester Blot was used to detect the interaction between Yap1 and TEAD4 after co-immunoprecipitation (Co-IP) was performed. **e**. Wetstern Blot was used to determine the expressions of CCNE1 and CCNE2 in T24 and Sw780 cells interfered by TEAD4-siRNAs

TEADs are best studied in the context of Hippo signaling and they are the primary transcription factors for the YAP/TAZ transcription co-activators. As the transcriptional co-activator, YAP cannot bind to DNA directly and usually YAP couples with TEADs to activate the transcriptional events. So, we speculated that Yap1 could be involved in the Cyclin E1/2 regulation at transcription level via binding with TEADs. First, we analyzed the basic expressions of TEAD1–4 in BLCA samples from TCGA database at transcriptional levels. The results showed that TEAD1 is under-expressed and TEAD4 is over-expressed in BLCA samples of the T group, compared with the normal (N) group (Fig. 4a). And there is a positive correlation between both TEAD1 and TEAD4 and Yap1 expression (Fig. 4b) at mRNA levels, which raises the possibility that Yap1 interacts with TEAD1 or TEAD4 in BLCA cells. Furthermore, we employed a nuclear complex co-immunoprecipitation technique to confirm the existence of the Yap1-TEADs interaction. In this assay, only TEAD4 was precipitated from the nuclear fraction by an anti-TEAD4 antibody and the presence of Yap1 in the precipitate was detected using anti-Yap1 antibody in the final immunoblot (Fig. 4d). But the interactions between Yap1 and TEAD 1, 2 and 3 were not detected in BLCA cells (data not shown here). All these results proved that Yap1 can interact with TEAD4 in T24 and Sw780 cells, here.

And the results from above, we have already known that metformin decreases the expression of CCNE1/2, CCND1, CDK4/6 and Yap1 and Yap1 may be involved in the regulation of these G1-phase related proteins in the BLCA cells. To elucidate the potential targets of Yap1, we examine the mRNA 111expressions of CCNE1/2, CCND1 and CDK4/6 as well as TEAD4 here in the T24 and Sw780 cells when interfered with Yap1-siRNAs. We found that Yap1-knockdown does not change the mRNA expressions of CCND1 (Additional file 2: Figure S2B), CDK4/6 (Additional file 2: Figure S2C and D) and TEAD4 (Additional file 2: Figure S2E), only the expressions of CCNE1 and CCNE2 have been obviously decreased at transcriptional level (Additional file 2: Figure S2F and G), which further increases the possibility of that Yap1 is closely related to the regulation of both CCNE1 and CCNE2 in the BLCA cells.

Besides these, we found that TEAD4 are positively correlated with the expressions of CCNE1 and CCNE2 (Fig. 4c) using the data from TCGA. To determine the possible involvement of TEAD4 in the regulation of CCNE1/2, we knocked down the TEAD4 expressions in the BLCA cells with RNAi method, too. And TEAD4 knockdown also results in the downregulation of both CCNE1 and CCNE2 at protein level (Fig. 4e), suggesting that TEAD4 is required for CCNE1/2 gene expressions in BLCA cells.

Taken together, all the results above have given us the hint that Yap1 could be involved in the regulation of CCNE1/2 expression by interacting directly with TEAD4.

### Yap1 interacts with TEAD4 directly regulating CCNE1/2 expressions at transcriptional level

**Fig. 5:**
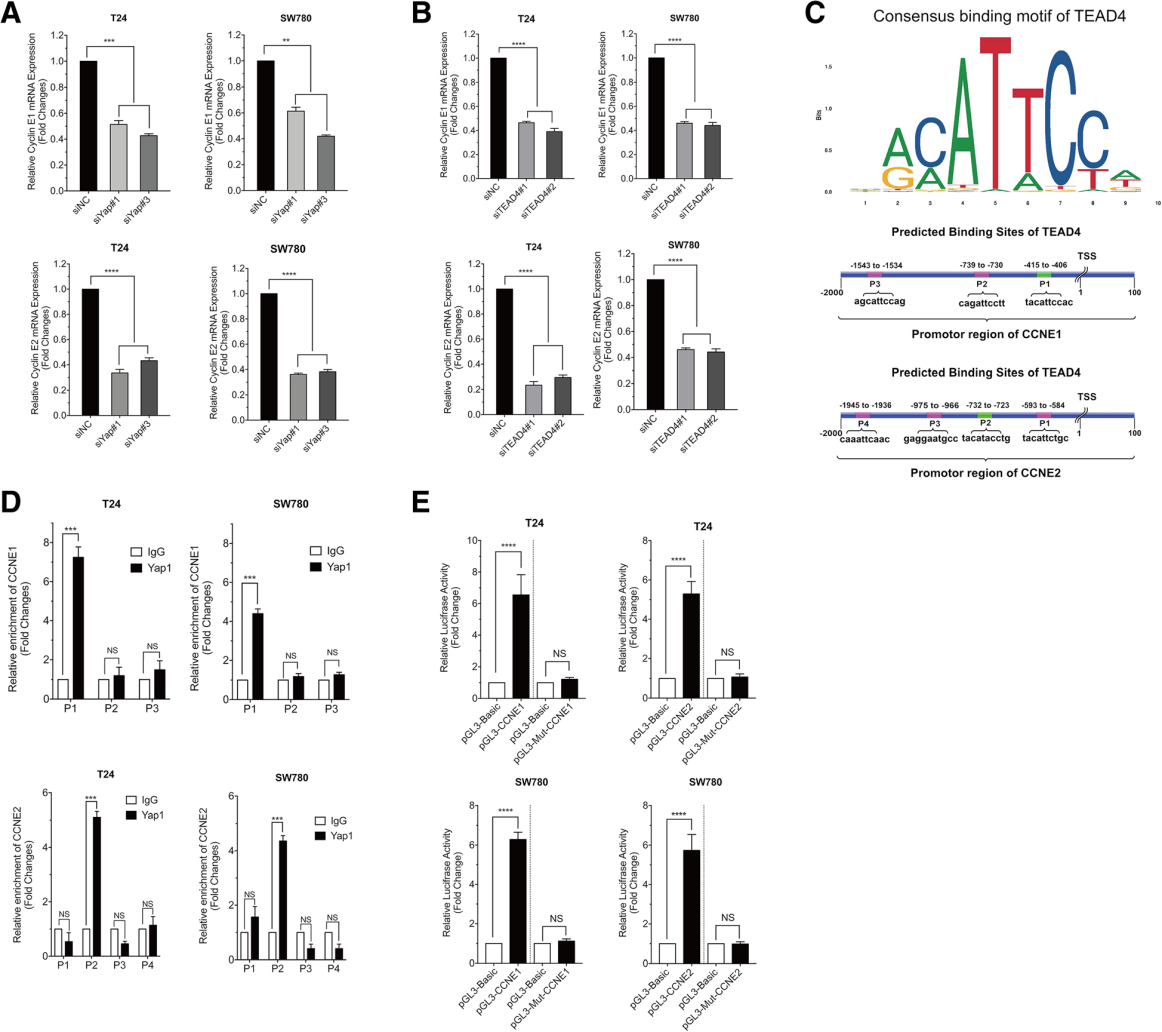
Yap1 regulates CCNE1 and CCNE2 gene expressions via TEAD4. **a** The relative mRNA expressions of CCNE1 and CCNE2 in T24 and Sw780 cells interfered with Yap1-siRNAs. **b** The relative mRNA expressions of CCNE1 and CCNE2 in T24 and Sw780 cells interfered with TEAD4-siRNAs. **c** Consensus binding motif of TEAD4 and the binding sites of TEAD4 on the promotors of CCNE1 and CCNE2 were predicted using JASPAR database. **d** ChIP-qPCR analysis was used to confirm the enrichment of CCNE1 and CCNE2 at the different binding sites predicted using Yap1antibody and a negative (IgG) antibody in T24 and 5637 cells. **e** Relative luciferase activities were determined in cell lysates for quantitation of CCNE1 and CCNE2gene expressions in T24 and Sw780 cells

To confirm the expression correlation between Yap1 and TEAD4 and the CCNE1/2 at transcriptional level in BLCA cells, we analyzed the mRNA levels of CCNE1/2, first, when Yap1 and TEAD4 were knocked down in the BLCA cells, respectively. As shown in Additional file 2: Figure S2F, G, Fig. 5a and Fig. 5b, both the knockdown of Yap1 and TEAD4 obviously inhibited the gene expressions of CCNE1/2, suggesting that Yap1 and TEAD4 could be the key regulator of CCNE1/2 expressions in the bladder cancer cells. Then, the JASPAR (http://jaspar.genereg.net) analysis of the TEAD4-occupied sites identified a consensus motif (Fig. 5b) and we found several TEAD4 binding sites located around − 1539 (P3), − 731 (P2) and -411 (P1) with respect to the TSS at the CCNE1 promoter; and around − 589 (P1), − 728 (P2), − 970 (P3) and − 1941 (P4) to the TSS site at the CCNE2 promotor with EPD (https://epd.vital-it.ch/master_search.php) and JASPAR database analysis (Fig. 5c).

Next, to determine whether Yap1 regulates transcription of CCNE1/2 directly via the formation of Yap1-TEAD4 complex in BLCA cells, we carried out the chromatin immunoprecipitation (ChIP) assay. We prepared two primer sets for the proximal promoter region of CCNE1 and CCNE2 genes to include the putative TEAD4 recognition motif. When precipitated with anti-YAP antibody, we detected positive PCR products of the proximal promoter regions of CCNE1 (at the P1 site relative to TSS) and CCNE2 (at the P2 site relative to TSS) respectively, although they were not detected in the other predicted regions (Fig. 5d). Ch-IP indicated that Yap1 could directly bind to the CCNE1 and CCNE2 promotor regions via TEAD4 in the BLCA cells. To provide further support for the notion that both CCNE1 and CCNE2 can be the direct target genes of YAP1-TEAD4 complex in BLCA cells, we used T24 and Sw780 cells to perform the luciferase reporter assay for the promoter regions of CCNE1 and CCNE2 genes. We found that the transduction of wild-type CCNE1 and CCNE2 promoter regions can show the enhancement of luciferase activity and the mutants in the promoter regions of CCNE1 and CCNE2 could not affect the luciferase activity (Fig. 5e).

These results have proved that Yap1 may bind with TEAD4 directly, playing a pivotal role in the transcriptional regulation of both CCNE1 and CCNE2 expressions in the BLCA cells.

### Effect of metformin on tumor growth in the BLCA xenograft model

The in vivo anticancer activity of metformin was investigated in a subcutaneous model of BLCA in which T24 cells (5 × 10^6^ mL^− 1^) were suspended and inoculated into the anterior axillary of healthy athymic nude mice. Previous in vivo xenograft studies [12, 17] reported that around 150 mg/kg/day - 250 mg/kg/day inhibited tumor growth and therefore, we used these dose in this study with the highly aggressive T24 cells. Metformin (150 mg/kg/day and 250 mg/kg/day) was administered daily for 14 consecutive days when the average tumor volume reached 100mm^3^.

**Fig. 6:**
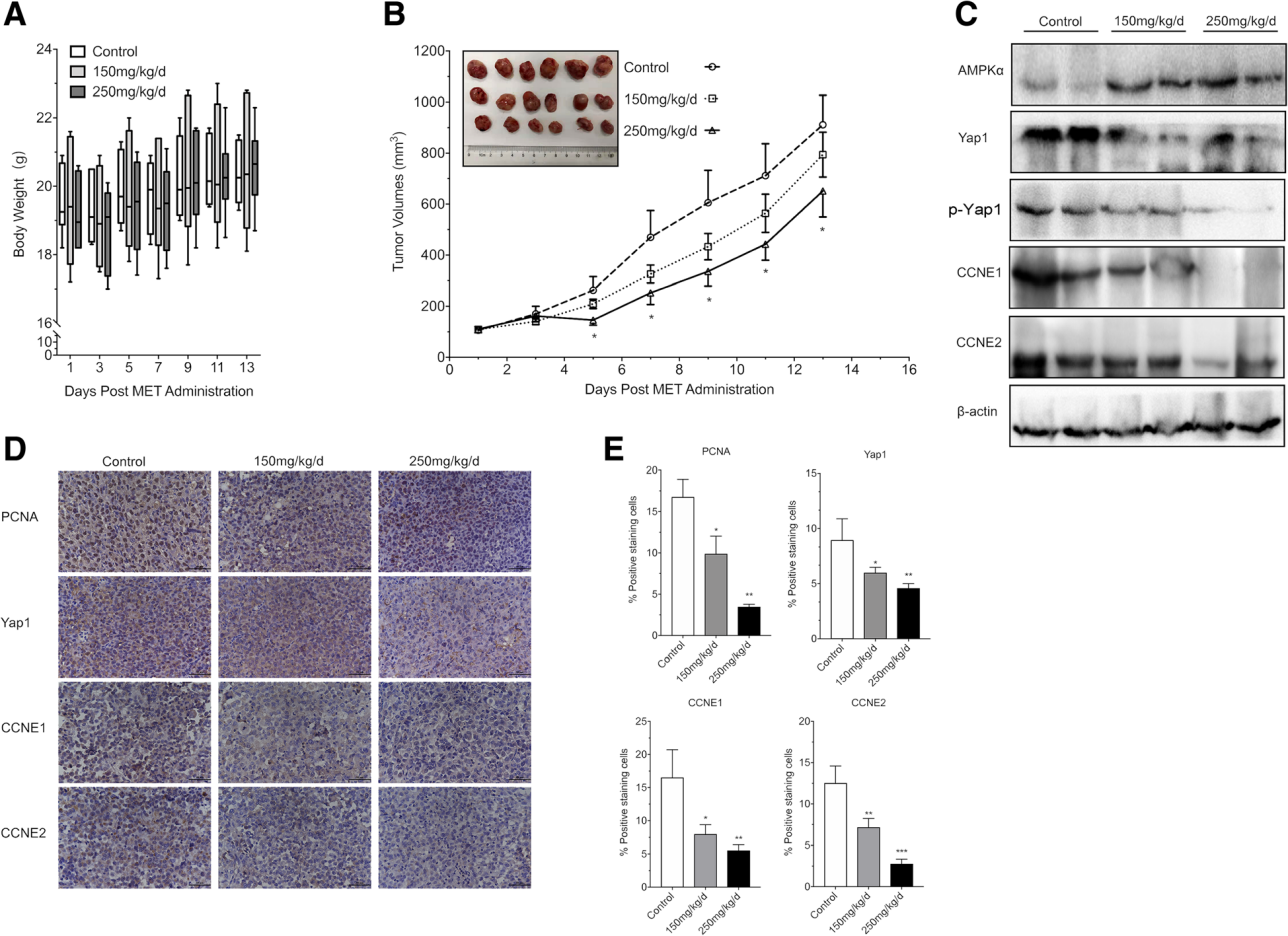
metformin inhibits the proliferation of BLCA cells in vivo. **a**. The total body weight of nude mice was measured when the treatment with metformin at the dosages of 150 mg/kg/d and 250 mg/kg/d was given. **b**. Tumor growth in xenograft mice receiving metformin treatment was recorded. **c**. The protein expressions of AMPK, Yap1 CCNE1 and CCNE2 was determined with Western Blot in the tumor tissues from the xenograft mice. **d**. The expressions of PCNA, Yap1, CCNE1 and CCNE2 were determined by Immunohistochemistry (IHC) staining derived from the xenograft. PCNA, Yap1, CCNE1 and CCNE2 positive cells were counted in 12 fields in each group. **e**. The percentage of PCNA, Yap1, CCNE1 and CCNE2 positive cells was calculated and presented as mean ± SD. All IHC analysis was performed with Image J software. * means *P* < 0.05 and ** means *P* < 0.01, compared to the control group

Once the mice were divided into treatment groups the body weight of each mouse was measured every 2 days for the remainder of the experiment. As shown in the Fig. 6 A, we found that metformin (at the doses of 150 mg/kg and 250 mg/kg body weight) did not change the body weights of mice during the therapeutic process. And the treatment with metformin at the dosage of 250 mg/kg significantly decreased tumor volume compared with control animals (Fig. 6b). Analysis of tumor lysates from control and metformin-treated mice showed an obvious increase in expression of phosphorylated AMPKa proteins; and there was a significant decrease in Yap1, CCNE1 and CCNE2 protein levels in tumors from mice treated with metformin (Fig. 6c). Moreover, immunohistochemical analysis (Fig. 6d) confirmed decreased staining of Yap1, CCNE1 and CCNE2 in tumors from the metformin treated animals. And as shown in Fig. 6e, the percentages of PCNA, Yap1, CCNE1 and CCNE2 positive cells are significantly lower in the metformin treatment groups than those in the control group.

Results of the in vivo and in vitro studies demonstrate that metformin-induced downregulation of Yap1 transcription co-factor also contributes to the anticancer activity of this antidiabetic drug.

## Discussion

Prior studies that have extensively reported the importance of metformin in cancer prevention and therapy. Functioning as an individual anti-tumor drug, metformin was reported to block precancerous lesions progressing to invasive tumors in bladder cancer [12]. And metformin efficiently suppressed the proliferation of bladder cancer cells in vitro and in vivo [36] and inhibited bladder cancer progression by inhibiting stem cell repopulation [37]. Besides these, metformin could be an excellent adjuvant for the combination treatment of bladder cancer. It was reported that metformin amplified and enhanced the effects of chemotherapy [13, 38]. Similar with these findings, our results showed that metformin alone obviously inhibited the proliferation of T24 and 5637 cells in vitro at supraphysiological conditions and xenograft models in vivo. To explore the possible reasons of metformin action, we performed cellular cycle and apoptosis analysis by Flow Cytometry. We found that the mechanism of growth inhibition could be due to cell cycle arrest at G1 phase, but without induction of cell apoptosis, which were also proved by the decreased expressions of CCNE1, CCND1, CDK2 and CDK4/6.

As a metabolic sensor, AMPK maintains cellular energy homeostasis and is a key player in a fast response of the cell to nutrient or energy [39]. Many findings suggested that AMPK is likely to exert tumor suppressor function, thus playing a critical role in cancer prevention [5, 40, 41]. Though there are many reports showing that AMPK can function as a tumor suppressive molecule, some other reports demonstrate the opposite. For example, it has been reported that AMPKα activation contributed to tumor growth in astrocytic tumors [42]. So, it is necessary to clear the impact of AMPK activation in different tumor types to avoid utilizing it as a therapeutic strategy in tumor types where it may promote growth.

At same time, it is well known that metformin is one of AMPK agonists and the resulting activation of AMPK is thought to be the main effector of the metformin anti-tumor activity. Here we found that metformin increases the phosphorylation level of AMPKα subunit in the BLCA cells and co-administration of AMPK inhibitor reverses the effects of metformin and the G1 arrest in the BLCA cells, hinting that AMPKα plays an important role of in metformin anti-tumor activity in BLCA cells. Recently, several different studies have also proved, from the other side, the contribution of AMPK activation as a mechanism for controlling bladder cancer growth by regulating proliferation [43–45] via different signaling pathways.

The evolutionarily conserved Hippo pathway is involved in the regulation of cellular proliferation, apoptosis, organ growth and tissue homeostasis. Most components of the Hippo pathway, especially the key downstream effector YAP1, has vital function in various human cancers. In the BLCA specifically, few studies addressed the relevance of Hippo signaling in bladder cancer. Until recently, there are some reports showing that the increased expression of Yap promoted cell growth and migration [46], and YAP inhibition inhibited the BLCA cell proliferation and restored sensitivity to cisplatin [47]. Furthermore, the crosstalk between YAP and Nrf2 played a critical role in bladder cancer chemoresistance [48]. YAP may serve as an oncogenic driver that confers cancer stem cell traits in bladder cancer [49]. These data suggest an important role of Yap in bladder cancer. We therefore assessed the effect of Yap1 activity on bladder cancer cells. We found that the AMPK activator metformin decreases the expression of Yap1 but without affecting its translocation from the cytoplasm to the nucleus in the BLCA cells in vitro. To confirm the role of Yap1 in the metformin-induced inhibition, we knocked down the expression of Yap1 with RNAi method. We found that Yap1 knockdown obviously blocks the cellular proliferation by inducing the G1 arrest in BLCA cells.

A series of intrinsic and extrinsic regulators are involved in the regulation of Hippo pathway and also are the cross-talk with multiple other signaling pathways. In reviewing the literatures, few studies reported the AMPK/Yap crosstalk. It was reported that AMPK induces YAP cytoplasmic retention by increasing the phosphorylation at Ser127 and inhibits its transcriptional activity [50], or AMPK directly phosphorylates YAP S94, a residue essential for the interaction with TEADs, thus disrupting the YAP-TEADs interaction [35]. In this study, we found that the AMPK activation induces the decreased phosphorylation levels of Yap1 as well as the degradation of total Yap1 protein in the BLCA cells, without causing its cytoplasmic retention. The degradation of total Yap1 can be restored by the addition of AMPK inhibitor CC, suggesting that AMPK activation directly promotes the ubiquitin-dependent degradation of Yap1 in the BLCA cells.

The accurate transition from G1 phase of the cell cycle to S phase is crucial for the control of eukaryotic cell proliferation, and its mis-regulation promotes oncogenesis. As the key regulator of G1-S transition, CCNE1/2 are also involved in the development of various cancers. The bioinformatics analysis showed that CCNE1 signature was an independent risk factor for BLCA and CCNE1 variants could be useful for BLCA risk prediction models [51–54]. We also found that CCNE1 gene expression is upregulated in the BLCA by searching the GEPIA Database. And we found that CCNE1/2 expressions are obviously decreased in the BLCA cell challenged by metformin and Yap1 knockdown can also inhibit its expression. So, we hypothesized that CCNE1 expression could be directly regulated by Yap1 in the BLCA cells. We all know that Yap1 is a transcriptional cofactor and exert its transcriptional regulatory functions through pairing with transcription factors, such as Smad, Runx2 and TEADs, to regulate transcription of a gene or set of genes. TEAD family includes TEAD1–4 in mammals, all of which share a highly-conserved DNA binding domain and YAP binding domain [55]. The interaction between YAP and all TEAD proteins has been confirmed both in vitro and in vivo, triggering the expression of Yap downstream genes involved in cell proliferation and survival. Here, we found that only TEAD4 is highly expressed and positively correlated with the expression of Yap1 in the BLCA cells via the bioinformatic analysis with GEPIA and Genevestigator Software. And the results of Co-IP assay showed that the anti-Yap1 antibody can successfully precipitate TEAD4 only in T24 and 5637 cells, which means that Yap1 directly interacts with TEAD4. TEAD4 knock-down also results in decreased expressions of CCNE1/2 in the BLCA cells. And the results of Luciferase Reporter Assay proved that Yap1 can directly regulate the activities of CCNE1/2 target genes via TEAD4. Similarly, it was reported that Yap1 formed complex with TEAD4 in the nuclei regulating the transcriptions of G1 arrest-related genes, such as CCND1, CCNE, CDK2, CDK4, and CDK6, in human oral squamous cell carcinomas [56]. And disrupting the YAP-TEAD interaction by verteporfin induced the cell cycle arresting at G1 phase via reducing the expression of cyclinD1 and cyclinE1 in pancreatic ductal adenocarcinoma [57]. Taken together, it is likely that treatment with metformin activates the AMPKα, which alters the Yap1/TEAD4/CCNE1/2 signaling, and induces the cycle arrest in the cells, which ultimately affects cell growth in the BLCA cells.

Most pre-clinical in vivo models have generally proven to be suboptimal for directing clinical application of new anti-cancer therapies, largely due to their inability to reflect the complexity and heterogeneity of human tumors. Our in vitro study was conducted using metformin at physiological levels of 150 mg/kg-250 mg/kg per day in our mice, which is equivalent to initial clinical dose of 500–1000 mg/daily in human [36]. Our in vivo study showed that metformin markedly inhibited the growth of human bladder tumor in a xenograft model. Given that the maximum recommended daily dose of metformin for the treatment of type 2 diabetic patients is around 2550 mg per day, our study indicates that metformin could exert its anti-cancer ability in vivo even at a safe dose level. But there are some limitations in our current study. Firstly, the inhibition effects of metformin on these BLCA cells are typically maintained in non-physiological conditions that are optimised only for in vitro growth and proliferation. As such, it is actually impossible to translate these supraphysiological conditions in the clinical setting, especially when the pharmacokinetics of the drug is different in culture settings and in the human body. Furthermore, we restricted the use of metformin to subcutaneous tumor models. Subcutaneous models are inferior to orthotopic models since the former do not represent appropriate sites for human tumors, and may not be accurately predictive for drug evaluation. Besides, the mechanism(s) by which metformin inhibits the proliferation of cancer cells is of diversity. Because there are many possible complicated cross-talks between the different signaling pathways in the cells, our findings are not the only ones that can be used to elucidate the precise mechanism, but the complementary to the others discovered. More research is required to plump the mechanism study of the inhibitory functions of metformin on the BLCA cells.

**Fig. 7:**
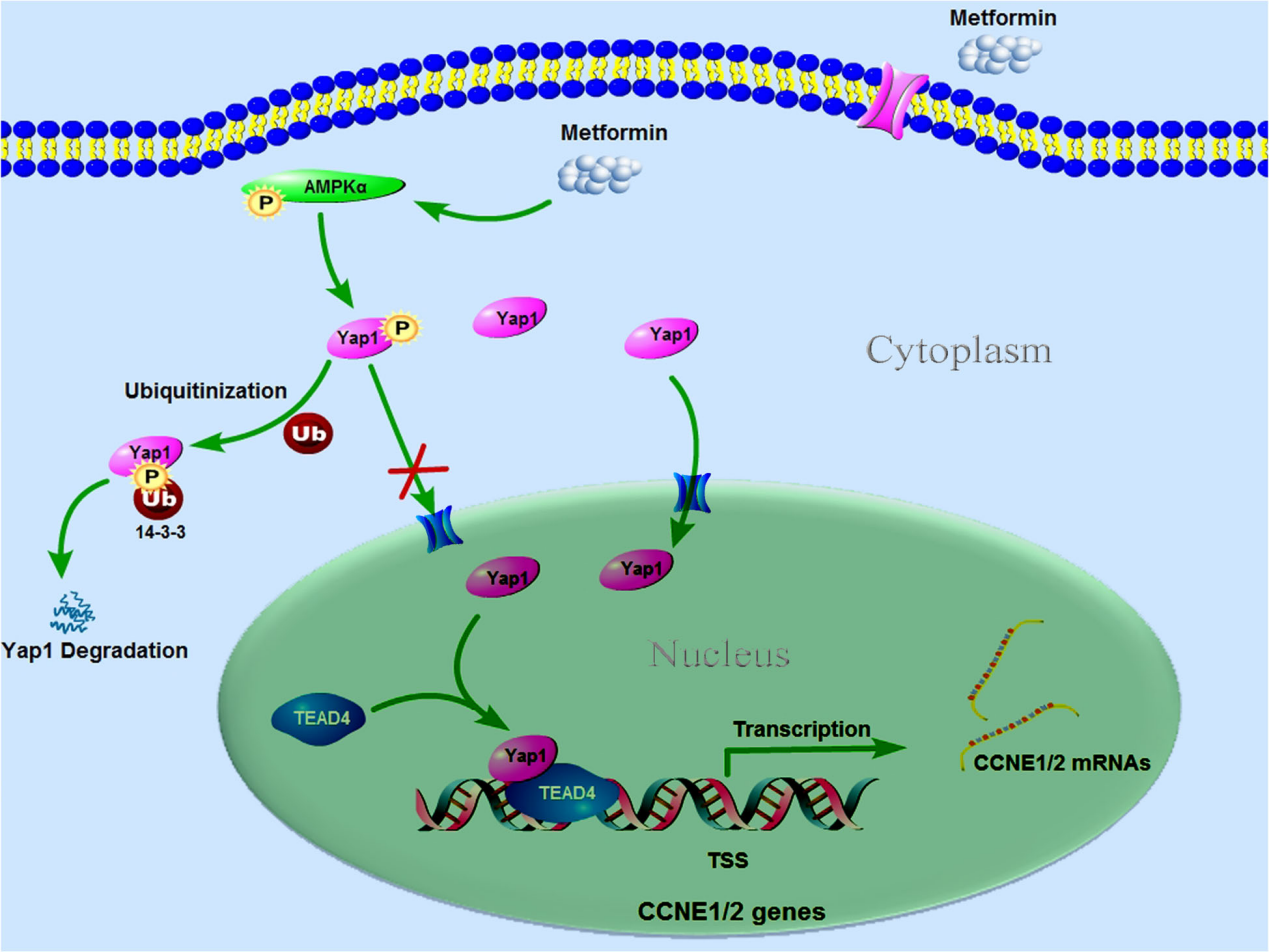
Scheme of CCNE1/2 suppression by metformin at transcription level via AMPKα/Yap1/TEAD4. Metformin activates AMPK by increasing the phosphorylation on its α subunit; and the activated AMPKα phosphorylates Yap1, resulting in its proteasomal degradation. Yap1 is involved in the regulation of both CCNE1 and CCNE2 via forming complex with TEAD4

In BLCA, Hippo signaling pathway is closely related to clinic-pathological characteristics and prognoses, which also has vital effects on the proliferation, metastasis and drug resistance of BLCA. As the key component of Hippo signaling, we therefore suggest that the Yap1/TEAD4/CCNE1/2 could be a potential therapeutic axis in BLCA (Fig. 7), via which metformin can be a potential candidate for the development of novel treatment strategies for human bladder cancer.

## Conclusions

In summary, our study reveals that the crosstalk between AMPK and Yap1/TEAD4 plays an important role in bladder cancer inhibition by metformin and that it is a critical cell cycle regulator that downregulates CCNE1 and CCNE2 directly. These findings will not only advance our understanding of the mechanism underlying cell cycle regulation and tumorigenicity, but also establish Yap1/TEAD4 as the key regulators of BLCA treatment and may also facilitate the development of new therapeutic strategies against BLCA. In essence, our study demonstrated the utility of metformin in assessing drug efficacy via molecular analysis and bioinformatics approaches. This is the first study that describes the concrete and direct molecular mechanism for the use of metformin in BLCA cells, providing direct evidence of metformin as an anti-cancer agent in BLCA.

## Additional files

Additional file 1: **Figure S1.** Metformin inhibits cell proliferation via inducing G1 cell cycle arrest in 5637 cells. A. Cell viabilities of 5637 cells were determined using MTS method when treated with metformin at different concentrations(5 mM, 10 mM and 20 mM) for at 24 h, 48 h and 72 h. B. Colony formation assay was carried out to evaluate the proliferation abilities of 5637 cells when challenged to metformin at different concentrations. C. The cellular apoptosis was analyzed with Flow Cytometry using Annexin V^+^ and PI^+^ staining in the 5637 cells treated with/without metformin at 48 h. D. Western Blot method was used to detect the expressions of Bcl-2 and Bax, cleaved Caspase 3 and 7. E. The cell cycle analysis was performed and compared with Flow Cytometry in T24 and Sw780 cells treated with/without metformin at 48 h. F. The key G1 phased related proteins, CCND1, CCNNE1/2 CDK4/6, and CDK2 were detected by Western Blot. * means *P* < 0.05, ** means *P* < 0.01, *** stands for *P* < 0.005 and **** stands for *P* < 0.001, compared to the control group. (TIFF 683 kb)

Additional file 2: **Figure S2.** Yap1 knockdown inhibits the mRNA expressions of CCNE1 and CCNE2. A. Expression of Yap1 was determined in the T24 and Sw780 cells transfected with Yap1-siRNAs. B. The relative expressions of CCNE1 were evaluated in T24 and Sw780 cells transfected with Yap1-siRNAs. C. The relative expressions of CDK4 were determined in T24 and Sw780 cells transfected with Yap1-siRNAs. D. The relative expressions of CDK6 were determined in T24 and Sw780 cells transfected with Yap1-siRNAs. E. The relative expressions of TEAD4 were determined in T24 and Sw780 cells interfered by Yap1-siRNAs. F. The relative expressions of CCNE1 were determined in T24 and Sw780 cells interfered by Yap1-siRNAs. G. The relative expressions of CCNE2 were determined in T24 and Sw780 cells transfected with Yap1-siRNAs. ** means *P* < 0.01, *** stands for *P* < 0.005 and **** stands for *P* < 0.001. (TIF 6604 kb)

Additional file 3: **Table S1.** Primers for ChIP-qPCR analysis of CCNE1 Promotor. **Table S2.** Primers for ChIP-qPCR analysis of CCNE2 Promotor. **Table S3.** Sequences of siRNAs. (DOCX 19 kb)

## Data Availability

The datasets used and/or analyzed during the current study are available from the corresponding author on reasonable request.

## Abbreviations

AMPK: Adenosine 5′-monophosphate (AMP)-activated protein kinase
BLCA: Bladder cancer
CC: Compound C
CCND1: Cyclin D1
CCNE1: Cyclin E1
CCNE2: Cyclin E2
CDKs: Cyclin-dependent kinases
EDTA: Ethylenediaminetetraacetic acid
FCM: Flow Cytometry
PCNA: Proliferating cell nuclear antigen
TEAD: TEA domain family member
TSS: Transcription start site
Yap1: Yes-associated protein 1
