# Supplementary figures and images for: Metformin targets a YAP1-TEAD4 complex via AMPKα to regulate CCNE1/2 in bladder cancer cells
Source: J Exp Clin Cancer Res. 2019 Aug 27;38:376. doi: 10.1186/s13046-019-1346-1 (PMC6712726; doi:10.1186/s13046-019-1346-1)

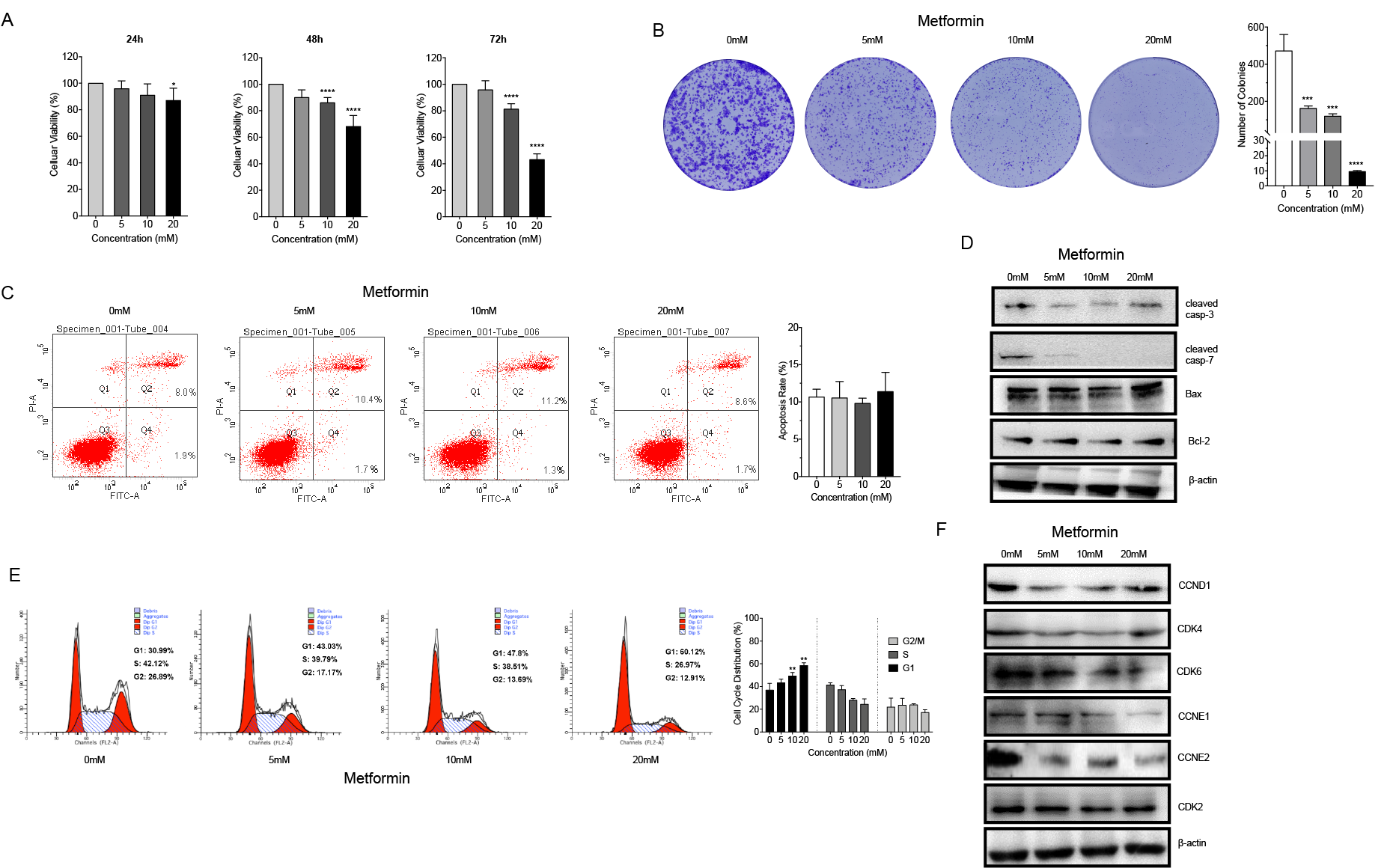

Supplement: Supplementary file 1 — Figure S1. Metformin inhibits cell proliferation via inducing G1 cell cycle arrest in 5637 cells. A. Cell viabilities of 5637 cells were determined using MTS method when treated with metformin at different concentrations(5 mM, 10 mM and 20 mM) for at 24 h, 48 h and 72 h. B. Colony formation assay was carried out to evaluate the proliferation abilities of 5637 cells when challenged to metformin at different concentrations. C. The cellular apoptosis was analyzed with Flow Cytometry using Annexin V+ and PI+ staining in the 5637 cells treated with/without metformin at 48 h. D. Western Blot method was used to detect the expressions of Bcl-2 and Bax, cleaved Caspase 3 and 7. E. The cell cycle analysis was performed and compared with Flow Cytometry in T24 and Sw780 cells treated with/without metformin at 48 h. F. The key G1 phased related proteins, CCND1, CCNNE1/2 CDK4/6, and CDK2 were detected by Western Blot. * means P < 0.05, ** means P < 0.01, *** stands for P < 0.005 and **** stands for P < 0.001, compared to the control group. (TIFF 683 kb) [file 13046_2019_1346_MOESM1_ESM.tiff]

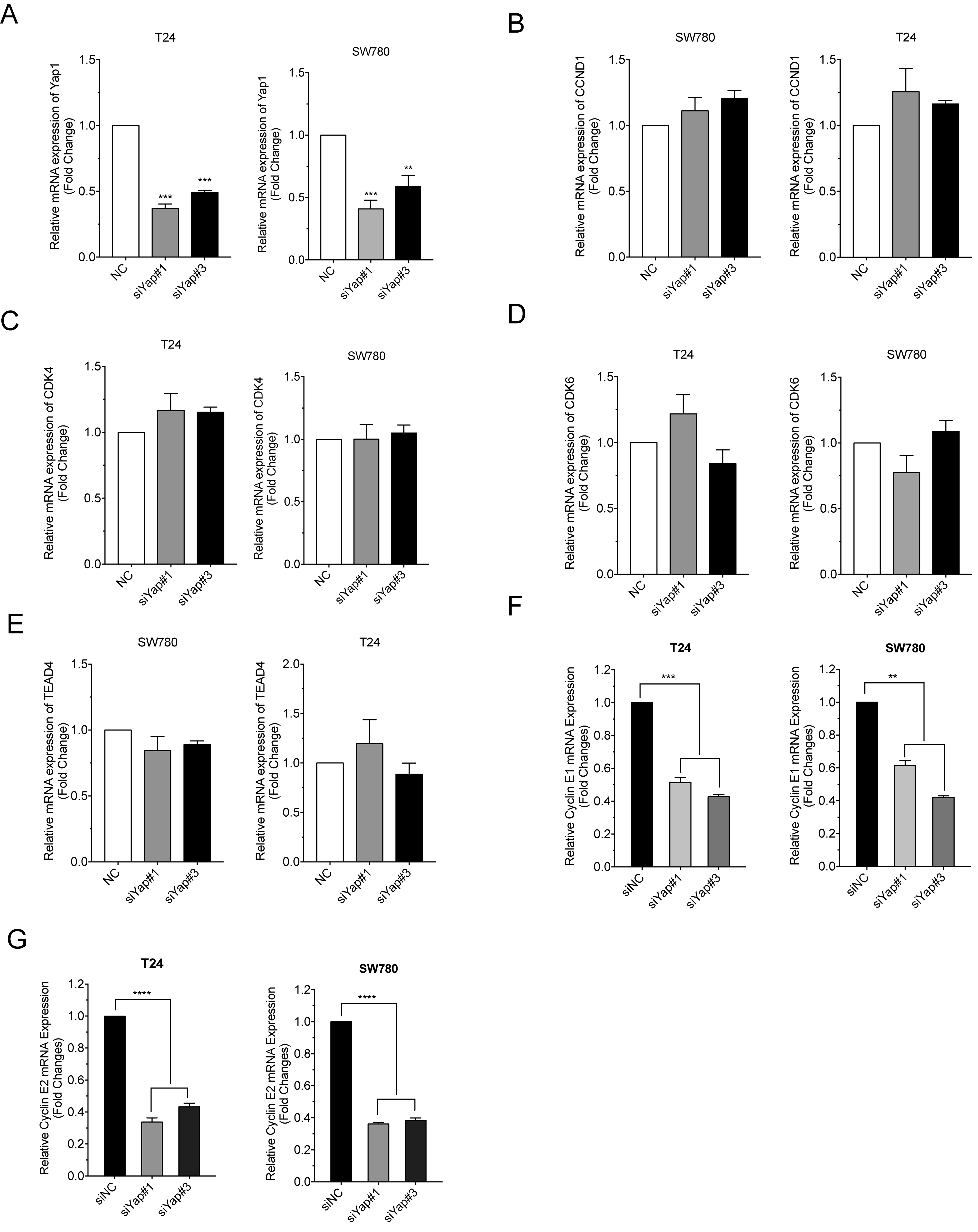

Supplement: Supplementary file 2 — Figure S2. Yap1 knockdown inhibits the mRNA expressions of CCNE1 and CCNE2. A. Expression of Yap1 was determined in the T24 and Sw780 cells transfected with Yap1-siRNAs. B. The relative expressions of CCNE1 were evaluated in T24 and Sw780 cells transfected with Yap1-siRNAs. C. The relative expressions of CDK4 were determined in T24 and Sw780 cells transfected with Yap1-siRNAs. D. The relative expressions of CDK6 were determined in T24 and Sw780 cells transfected with Yap1-siRNAs. E. The relative expressions of TEAD4 were determined in T24 and Sw780 cells interfered by Yap1-siRNAs. F. The relative expressions of CCNE1 were determined in T24 and Sw780 cells interfered by Yap1-siRNAs. G. The relative expressions of CCNE2 were determined in T24 and Sw780 cells transfected with Yap1-siRNAs. ** means P < 0.01, *** stands for P < 0.005 and **** stands for P < 0.001. (TIF 6604 kb) [file 13046_2019_1346_MOESM2_ESM.tif]
