# Supplementary material for: Metformin targets a YAP1-TEAD4 complex via AMPKα to regulate CCNE1/2 in bladder cancer cells
Source: J Exp Clin Cancer Res. 2019 Aug 27;38:376. doi: 10.1186/s13046-019-1346-1 (PMC6712726; doi:10.1186/s13046-019-1346-1)
Supplement: Supplementary file 3 — Table S1. Primers for ChIP-qPCR analysis of CCNE1 Promotor. Table S2. Primers for ChIP-qPCR analysis of CCNE2 Promotor. Table S3. Sequences of siRNAs. (DOCX 19 kb) [file 13046_2019_1346_MOESM3_ESM.docx]

| Position | Primers for CCNE1 |
| --- | --- |
| P1 | CGCCGTGTTTACATTCCACCCG GGACGCGGGAGAAGTCTGGC |
| P2 | AATCCCAGAGTCAGAAAGGTCTTCAGA GCCTGTCCATTCATCCGTCAGTG |
| P3 | TGGGAGCATTCCAGAGCCTTCT GGAACAGTCATCACCTCAGAGTACC |

Additional file 3

Table S1, Primers for ChIP-qPCR analysis of CCNE1 Promotor

Table S2, Primers for ChIP-qPCR analysis of CCNE2 Promotor

| Position | Primers for CCNE2 |
| --- | --- |
| P1 | GTGGCTGAGTGGTGTTTACATTCTG GAGCAAAGCGTTGGCTGAAGAG |
| P2 | GGCAGAGCGGCGGGTTGG CGCCCACCCAGGTATGTATCAAC |
| P3 | GTGGGCGGGCTGGAAATGC CCAAGTGCGGCTCTGCTCTC |
| P4 | TCCCACAAACGTTACTCGACATACTG GTCACCTGATCGTAAGCAGAACTGG |

Table S3, Sequences of siRNAs

| Names | sequence |
| --- | --- |
| siNC | UUCUCCGAACGUGUCACGUTT  ACGUGACACGUUCGGAGAATT |
| siTEAD4#1: | CUGUGCAUUGCCUAUGUCUdTdT  AGACAUAGGCAAUGCACAGdTdT |
| siTEAD4#2: | CAGAGUAUGCUCGCUAUGAdTdT  UCAUAGCGAGCAUACUCUGdTdT |
| siTEAD4#3: | GUGGACAUCCGCCAAAUCUdTdT  AGAUUUGGCGGAUGUCCACdTdT |
|  | |
| siYap1#1: | GCUCAUUCCUCUCCAGCUUdTdT  AAGCUGGAGAGGAAUGAGCdTdT |
| siYap1#2: | CUCUUCAACGCCGUCAUGAdTdT  UCAUGACGGCGUUGAAGAGdTdT |
| siYap1#3: | CACCUAUCACUCUCGAGAUdTdT  AUCUCGAGAGUGAUAGGUGdTdT |
